# Idiopathic Normal Pressure Hydrocephalus: The Real Social and Economic Burden of a Possibly Enormous Underdiagnosis Problem

**DOI:** 10.3390/tomography9060157

**Published:** 2023-10-30

**Authors:** Gianpaolo Petrella, Silvia Ciarlo, Stefania Elia, Rita Dal Piaz, Paolo Nucera, Angelo Pompucci, Mauro Palmieri, Alessandro Pesce

**Affiliations:** 1 Neurosurgery Division, A.O. “Santa Maria Goretti” General Hospital, Via Guido Reni 1, 04100 Latina, Italy; gianpaolo_p@hotmail.com (G.P.); silvia.lt@hotmail.com (S.C.); elia.1385392@studenti.uniroma1.it (S.E.); angelo.pompucci@gmail.com (A.P.); 2 Emergency Department, A.O. “Santa Maria Goretti” General Hospital, Via Guido Reni 1, 04100 Latina, Italy; r.dalpiaz@ausl.latina.it; 3 Emergency Department, A.O. “Dono Svizzero” General Hospital, Via Appia Lato Napoli, 04023 Formia, Italy; p.nucera@ausl.latina.it; 4 Department of Human Neurosciences, Neurosurgery Division, Università “La Sapienza” di Roma, Viale del Policlinico 155, 00161 Roma, Italy; mauro.palmieri10@gmail.com

**Keywords:** idiopathic normal pressure hydrocephalus, iNPH, CT scan, misdiagnosis, underdiagnosis, lumbar infusion test

## Abstract

Normal Pressure Hydrocephalus (iNPH) typically affects the elderly and can cause cognitive decline, resulting in its differential diagnosis with other neurodegenerative conditions. Moreover, it is probably underdiagnosed; such under- and misdiagnosis prevents the patient from receiving the right treatment and significantly affects the quality of life and life expectancy. This investigation is an in-depth analysis of the actual incidence of iNPH in the population of the province served by our hospital (circa 580,000 individuals). The first phase of this study was conducted by visualizing a total of 1232 brain CT scans performed in the Emergency Departments of the four hospitals of our network on patients who were admitted for different complaints yet screened as suspicious for iNPH. Subsequently, corresponding Emergency Department medical records were investigated to understand the medical history of each patient in search of elements attributable to an alteration of CSF dynamics. The cohort of positive CT scans, according to the radiological and clinical inclusion criteria, included 192 patients. Among the reasons to require acute medical care, “Fall” was the most common. The cumulative incidence of CT scans suggestive of iNPH among the patients undergoing CT scans was as high as 15.58%, and the period prevalence calculated for the total amount of patients accessing the Emergency Departments was 1.084%. The real incidence of iNPH in the population may be underestimated, and the social burden linked to the assistance of patients suffering from such untreated conditions could be significantly relieved.

## 1. Introduction

### 1.1. Background

Idiopathic normal pressure hydrocephalus (iNPH) is a condition linked to an increase in the size of the ventricular cavities, without an equivalent increase in CSF pressure in the intrathecal compartment. It was described for the first time in 1965 by Hakim and Adams, who observed a neurological condition that would later be defined as the Hakim–Adams triad, composed of progressively worsening cognitive impairment, urinary incontinence, and gait disturbances [1]. It is a typical condition of advanced age; its prevalence is estimated at around 1.3% in patients over the age of 65 [2,3]. Such an elusive condition may, however, be burdened by the critical problem of under- and misdiagnosis. iNPH, despite being one of the first conditions to be taken into account in the process of the differential diagnosis of neurocognitive deterioration, often remains misdiagnosed or even ignored in community health systems, which are in charge of the very first evaluations of patients suffering from cognitive decline [4].

Critical landmarks of the diagnosis are both the neurological and general evaluation of the patient as well as the execution of non-invasive and invasive instrumental examinations: among the first, we include computed tomography (CT) and magnetic resonance imaging (MRI), through which the classical ventricular dilatation indices are easily calculated, such as, for instance, the Evans index, (>0.3), Callosal angle (positive if <80° [5]), and DESH (disproportionally enlarged subarachnoid spaces and hydrocephalus) [5,6,7,8,9]; among the invasive tests, we perform the CSF withdrawal test or lumbar tap test as well as the lumbar infusion test with real-time intrathecal pressure measurement [10,11,12,13,14]. The mainstay of treatment for normal pressure hydrocephalus is the implantation of a ventriculoperitoneal shunt system, which allows for the drainage of the cerebrospinal fluid from the ventricles into the peritoneal cavity [10]. The success rate of surgery ranges between 70 and 90% [4]—higher in younger patients—with improvement in cognition, walking, and urge incontinence [12].

### 1.2. Objective

Such a condition may still be critically underdiagnosed [13,14,15,16,17], especially in community medicine centers located in remote areas. Because some basic radiological investigations, such as a simple brain CT scan, coupled with the analysis of the medical records of the patients could produce reliable information concerning the possible presence of NPH symptoms, it may finally be possible to shed light on the real number of NPH patients daily accessing our Emergency Departments, thus resulting in a step further in understanding the real incidence of such a condition in the elderly.

## 2. Materials and Methods

### 2.1. Participants and Study Setting

This investigation was strictly focused on the territory of our wide community health system (Provincial Health System), which extends for 2256 km^2^, including a total of 576,655 inhabitants comprising 285,153 males and 291,502 females. The population of Latina is characterized by a relatively young population, with an average age of 43.8 years—the youngest recorded in our region, Lazio. Such a wide system comprises five major hospitals, among which just one has a Neurosurgical division and is de facto in charge of the neurosurgical patients of the province.

### 2.2. Inclusion and Exclusion Criteria

We divided the inclusion and exclusion criteria used into two categories:

Clinical: we included all patients

- over the age of 65;
- who had access to the Emergency Departments of the province of Latina;
- and whose personal data and medical history data can be found in our electronic medical records archive.

Radiological: we included all patients

- who had an obvious increase in the size of the ventricles;
- and in whom other intracranial pathologies were excluded; in order to avoid interference on the CSF dynamics in particular, we excluded all patients who had a previous or concurrent history of intracranial bacterial, viral, or fungal infections—and of course, any intracranial hemorrhage.

Only if all of the aforementioned clinical and radiological criteria were matched were the patients enrolled in this investigation.

### 2.3. Data Sources and Investigated Variables

With the aid of our radiological PACS system (which is the digital archive containing all the radiological investigations carried out in the hospital of our province), we reviewed brain CT scans performed on patients accessing the Emergency Departments (ED) of the Latina, Formia, Terracina, and Fondi Hospitals, taking into consideration a specific period of 30 days. We viewed a total of 1232 CT scans and selected 192 scans from patients who met our inclusion and exclusion criteria and whose scans, according to our evaluation, resulted in suspicion for iNPH.

Finally, we consulted ED medical records in search of elements leading to the diagnostic suspicion of iNPH in the clinical records of our Emergency Department and the neurological and general evaluation of each patient.

Precisely and from a radiological perspective, we specifically investigated the following for each of the CT scans:

1. The callosal angle as measured at the coronal slice depicting the Monro foramina, suspicious if ≤80°. This was recorded both as a dichotomous variable (0/1->80°/<80°) and as a continuous variable, namely, the exact angle measured;
2. The presence of a possible disproportion between the subarachnoid spaces of the basal cisterns and of the vertex sulci (DESH, Disproportionately Enlarged Subarachnoid Space Hydrocephalus). This was recorded as a dichotomous variable (0/1–absent/present);
3. Evan’s index, measured as the maximal frontal horn ventricular width divided by the wider inner transverse intracranial diameter (ventriculomegaly if it was 0.3 or greater). This was recorded both as a dichotomous variable (0/1-<0.3/>0.3) and as a continuous variable, namely, the exact Evan’s Index measured;
4. Enlarged intracranial ventricles. This was recorded as a dichotomous variable (0/1–absent/present).

As specified before, the presence of other intracranial space-occupying neoplastic, infectious, or hemorrhagic conditions and the presence of CSF outflow pathologies were obvious exclusion criteria.

### 2.4. Outcome Measures

Lastly, we calculated the period prevalence of such suspicious CT scans, concerning the total number of brain CT scans performed in that period of 30 days, and the incidence for the global population who accessed the ER of our hospital network during the examined week.

We used period prevalence, because we considered a precise “time interval” of 30 days: in such an interval, for the purposes of this investigation, the conditions in which the clinical and radiological landmarks of a “suspected” diagnosis were present were considered “cases” (or more precisely, “positive events”). We used the following formula:

*Period Prevalence of iNPH* = *number of existing clinically and radiologically suspected patients in a certain period/number of total patients requiring a CT scan in that same period.*

We then completed the estimation by calculating the cumulative incidence rate in order to obtain the number of “new suspected diagnoses” in the general population accessing the Emergency Room in our period of investigation. The general population of the individuals accessing the Emergency Department in that specific month was a known number, considered a “closed population”. We used the following formula:

*Cumulative estimated incidence* = *number of new suspected cases/number of individuals accessing the Emergency Room for any reason.*

### 2.5. Statistical Methods and Potential Sources of Bias

The sample was analyzed with SPSS version 18 (IBM, Chicago, USA). Residual analyses of nominal and dichotomous variables were made with Chi^2^ and Binomial residual analysis tests, respectively. Continuous variable correlations were investigated with Pearson’s Bivariate correlation, whereas Spearman’s method was used for ordinal variables. Incidence and prevalence were calculated based on the digital record system active in our institutions. The threshold of statistical significance was considered *p* < 0.05.

We addressed no missing data since incomplete records were an exclusion criterion. A potential source of bias was to be expected from the exiguity of the sample and from the limitedness of the time on which the investigation focused. The informed consent was approved by the Institutional Review Board of our institution. Data retrieved from the general radiological and ER clinical archives were anonymized, with exceptions taken for sex, age, hospital of access, and clinical complaints that required acute medical care. Data reported in this study have been completely anonymized. No treatment randomization was performed. No data apart from clinical and CT data were examined. This study is consistent with the Italian regulation concerning Data Management and Privacy (D.L. 10 August 2018 n. 101; it is a retrospective archival anonymized non-randomized investigation) and the Helsinki Declaration of Human Medical Research.

## 3. Results

**Figure 1:**
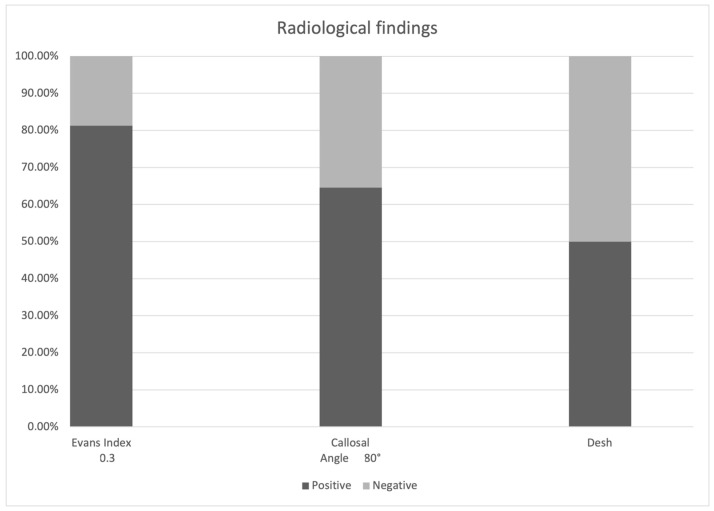
Bar graph summarizing the radiological findings.

In the final cohort, composed of 192 patients (Table 1), a total of 81.25% (*n* = 156) of the patients presented an Evans index greater than or equal to 0.3 (Binomial non-parametric analysis *p* = 0.0001); furthermore, we found a reduced callosal angle below 80 degrees in 64.58% of the patients (Binomial non-parametric analysis *p* = 0.059), while DESH was positive in 50% of the cases. Most of these patients, therefore, presented morphological ventricle alterations (further details in Figure 1).

We then analyzed the reports of these CTs; 41.7% of these tests were given as negative. Ventricular dilatation was reported in the radiological report in just 33.3% of the cases (64/192 cases, Chi^2^ residual analysis *p* = 0.029), as well as cortico–subcortical atrophy. Interestingly, the word “hydrocephalus”, even in cases in which all the three aforementioned radiological landmarks were present, was never reported. The rationale of the CT scans of course played a significant concurrent role: the radiologists paid more attention to rule out emergent conditions. For instance, in the first week, of the 56 CTs performed on 8 March 2022, nine were positive. Of these, two were in Latina, one was in Fondi, four were in Formia, and two were in Terracina.

**Figure 2:**
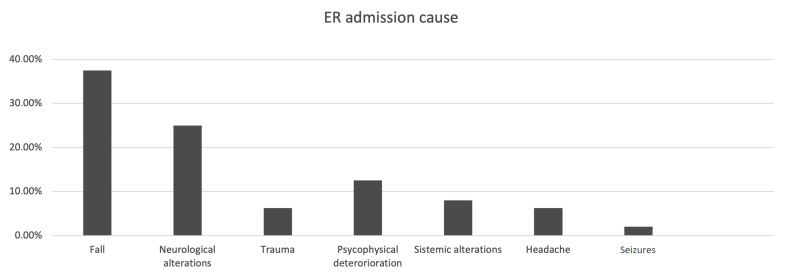
Bar graph summarizing the causes for ER admission.

From a clinical perspective, in Figure 2, we divided the patients according to the reason for their access to the ED; it is remarkable that 38.02% of the patients accessed the ED due to a head trauma related to a fall. Frequent falls are often one of the first clinical signs in patients later diagnosed with iNPH because of gait disturbance. Other factors that could lead to clinical suspicion are cognitive impairment, present in 13.02% of patients; headache in 6.25%; and even status epilepticus, which can present itself at onset, albeit rarely. The history of “Falls” was significantly more frequent than the other causes (Chi^2^ residual analysis 72/192 for −6.7 residuals, *p* = 0.0001).

In Figure 2, details concerning the neurological evaluation are reported in detail. The examination was negative in 57.81% (111/192) of the patients, whereas neurological focal deficits were identified in 15.10% (29/192) of the patients. An obvious state of cognitive impairment was reported in 21% (40/192) of the patients. A possible bias could be given by this omission: that in fact, dementia in elderly patients may be taken for granted, or at least considered a non-emergent condition and therefore not reported.

### Main Results

The final calculation regarded the period prevalence of the positive CT scans concerning the total number of CT scans performed in the examined month: the amount of CT scans suspicious for iNPH is 192 positive CT scans divided by the total amount of 1232 examined brain CT scans in the same period for a cumulative incidence of 15.58% when considering the specific population of patients undergoing brain CT scans in that specific period. From the general register of our Province Health System, we had a total of 17,696 patients accessing the Emergency Departments of our hospitals in that same month for any reason, which amounted to an overall period prevalence of 1.084% of the general population requiring acute medical care.

## 4. Discussion

Idiopathic Normal Pressure Hydrocephalus (iNPH) is a condition linked to the dilatation of the intracranial ventricular cavities without an obvious increase in intracranial pressure [1,2]. Recognized risk factors include ethnicity; cardiovascular concurrent conditions such as arterial hypertension, cholesterol, and glucose levels [18,19,20]; obesity; a history of alcohol abuse; psychosocial issues [20]; and even psychiatric disorders [20,21]. The pathogenesis is not perfectly clarified but most likely to be multifactorial [21,22,23]; several investigations among these factors included increased CSF pulsatility, reduced CSF outflow, alteration of the blood–brain barrier, focal ependymal hypoperfusion, and even neuroinflammation processes, as well as glymphatic system disturbances. With the increasing average age of the population and with improved accessibility to diagnostic tools, incidences are constantly growing [2,23]; therefore, it should be considered a constant “hot” topic in neurosurgery.

The most complex feature of the iNPH clinical practice is its difficult diagnosis [13,14,23], which is rather elusive and for which maneuvers such as tap tests, CSF subtraction tests, and intrathecal infusion tests have been proposed. In previous investigations [13,14], we discussed the possible decisive role played by the lumbar infusion test to confirm diagnosis with a notable sensitivity and specificity level, especially when combined with a specific set of pre-and postoperative neuropsychological tests [14].

Even when making use of the precious information derived from the lumbar infusion test, MRI imaging appears to be an unavoidable milestone in the diagnosis of iNPH. Such imaging modalities are able to confirm the enlargement of the ventricular system, demonstrating the classical signs of DESH; the presence of periventricular edema (transependymal resorption); and the “flow void sign” [24]. Further studies outlined the possible clues that indicated an increased cerebral stiffness via performing an MRI elastography [25] and possible alterations of the cerebral blood flow as investigated with an MR Perfusion study [26].

In the Emergency Room setting, an MRI is rarely or even never performed to address the clinical and CT suspicions of iNPH. This investigation focuses on the first-line examination that could raise suspicions for an iNPH diagnosis; in fact, as previously demonstrated [27], the prompt recognition of this syndrome and earlier treatments such as ventriculoperitoneal shunting are associated with better outcomes, thus possibly ameliorating the overall prognosis of this group of patients and reducing the social and economic burden of iNPH. Moreover, on one hand, a first-line CT scan demonstrates ventricular enlargement, the value of the Evan’s Index, and the callosal angle [28], thus providing a strong clue to an iNPH; on the other hand, it serves as a lesson learned: the path between the first CT scan (with a negative report) and the subsequent MRI scan can be all but straight.

In fact, over years of direct experience in our territory, we developed the idea of a profound under- and misdiagnosis of this condition, which is, by the way, shared globally by different research groups [4,29]; for this, several factors could be advocated as causative—for instance, limited access to full medical care in remote areas and a limited awareness of such a condition by the community physician active in the aforementioned areas.

Survey studies derived from Japanese nation-wide research groups, as well as wide Norwegian and Swedish cooperative researchers [24,28,29], highlight a possible crude prevalence as high as 5.5–21.9/100,000 individuals. Our results are similar. This is of uttermost importance when we consider that iNPH is one of the few really reversible causes of cognitive decline [24]. The historical results of Conn et al. [30] are remarkable: up to one third of the physicians who returned the survey had never heard of iNPH!

Our findings point out that an estimated overall period prevalence could be as high as 1.084% in a closed population of patients requiring acute medical care, and this could be catastrophically underestimated. Apart from the natural and unavoidable biases that such an incidence/prevalence investigation presents, such results appear to be impressive and remarkable, especially when considering that 41.7% (80/192 patients) received negative radiological reports and in just 33.3% (64/192 patients), ventricular enlargement was recognized and deemed to be worth mentioning.

Radiological findings aside and from a purely clinical perspective, the Emergency Room evaluation performed by emergency physicians could focus on acute complaints, thus producing a further underestimation of the three clinical complaints classically defined as the Hakim “triad” [1]: namely, cognitive decline, gait disturbance, and incontinence. Among the clinical records of the investigated patients, we were able to retrieve “Fall”, “Trauma”, and “Neurological deterioration”, which could theoretically be traced back to iNPH, but we did not find a single precise mention of the Hakim triad, which still plays a role in the medical history recollection of iNPH patients.

Accordingly, availing ourselves of the support of the local medical council, we developed a series of meetings and congresses to ameliorate the knowledge and understanding of iNPH, thus increasing the sensitivity of the “first evaluation” physicians who are active in community health centers with an increasing number of treated patients [31,32]. The impact of this strategy is currently being evaluated in other investigations.

### 4.1. Main Results and Interpretation

In this paper, we wanted to specifically focus on the social and economic burden of undiagnosed iNPH. From a purely economic perspective, which is a key perspective in countries like Italy, in which healthcare is fully covered by public national health systems, recent calculations (which were included in the public health cost calculations for the PNRR, Italian Plan for resilience and recovery) determined that for each patient suffering from neurodegenerative diseases like Alzheimer disease or iNPH, the disease-related costs amount up to EUR 70,580 a year [33].

Only in our territorial competence and in a period of just 30 days, the total amount of patients who underwent a first-level diagnostic with results suspicious for iNPH is as high as 192; we can therefore state that this is equivalent to circa 2300 patients in one year. This calculation is restricted to patients who required acute medical care in the network of our hospitals; therefore, it further suffers from an unavoidable underestimation bias. The resulting total expenditure therefore amounts to numbers as high as EUR 169,320,000 a year, an impressively high cost when compared to the much lower average cost for a ventriculoperitoneal shunt surgery, which ranges around EUR 12,891 (which is the exact evaluation of the hospitalization-related costs in our region, Lazio [33]), for a cumulative resulting public cost of EUR 30,938,400. If these patients were diagnosed with iNPH, a total amount of EUR 138,381,600 would be saved in the first year for each patient and then resolved for subsequent years. Similar results were previously reported in a slightly different fashion in another paper [34]: Tullberg et al. demonstrated that iNPH patients, undergoing a successful shunt surgery, would benefit from 2.2 additional life years and 1.7 years with a lower personal healthcare burden.

Of course, adverse events related to ventriculoperitoneal shunting involving typical complications such as infection, malposition, and overdrainage bring further unjustified costs that weigh over the National Health System; in an interesting investigation conducted in the USA, non-routine discharges and emergent admission significantly prolonged the length of the stay and treatment-related costs [35]. Therefore, even with regard to public health-related costs, the focus unquestionably remains on a precise diagnostic protocol and on a correct surgical indication.

### 4.2. Limitations and Future Perspectives

This investigation has several limitations: the calculations concern a limited amount of time; furthermore, the initial cohort of patients is derived from a group of individuals belonging to a precise area of Central Italy who specifically required acute medical attention in one of our hospitals and who did not directly elect to undergo specific neurological tests for neurodegenerative conditions. All these features could lead to an underestimation bias of the total number of patients suffering from an undiagnosed iNPH. To validate the presented data, each of these patients will be contacted via telephone in an upcoming investigation and will undergo a simple questionnaire to deepen the preliminary evaluation and screen those whose clinical history requires an in-depth evaluation to rule out or disclose iNPH. Subsequently, the patients presenting a “suspicious” pattern of symptoms and a “suspicious” brain CT scan will undergo an accurate brain MRI scan; a lumbar infusion test to estimate the pressure, elastance, and compliance of the intrathecal compartment; and an accurate neuropsychological evaluation, which will be performed, as previously reported [14], using three tools calibrated for the Italian population: the Frontal Assessment Battery, Mini Mental Status Evaluation, and Montreal Cognitive Assessment (MOCA). This complex evaluation system proved to be promising in a previously published series of patients. Despite all the considered biases, the iNPH underdiagnosis problem could be critical, even according to our data.

## 5. Conclusions

Normotensive hydrocephalus is a complex, high-incidence, and probably underdiagnosed disease. Patients may experience significant iNPH-related complications, leading to a notable reduction in their quality of life and life expectancy. The personal and social burden of such a complex disease could be reduced with the increased knowledge of this condition, which could allow for a more accurate and timely diagnosis, eventually leading to a technically simple surgery with few complications and a decisively ameliorating impact on the quality of life.

## Figures and Tables

**Table 1:** Patient’s demographics.

	N = 192 Patients
Sex	Male N = 106 − 55%
	Female N = 86 − 45%
Age	82.1 years ± 10.9
Reason of Emergency Room admission	Fall 37.50% (72/192 patients)
	Neurological deficit 25.00% (48/192 patients)
	Trauma 25.00% (48/192 patients)
	Cognitive impairment 12.50% (24/192 patients)
Clinical manifestations	None 59.37% (114/192 patients)
	Neurological deficit 15.7% (30/192 patients)
	Cognitive impairment 20.83% (40/192 patients)
	Seizure 1.57% (3/192 patients)
	Astenia 3.12% (6/192 patients)
Evans index ≥ 0.3	81.25% (156/192 patients)
Callosal angle ≤ 80°	64.58% (124/192 patients)
DESH	50.00% (96/192 patients)
Cortical atrophy	32.81% (63/192 patients)

DESH: disproportionally enlarged subarachnoid spaces and hydrocephalus.

## Data Availability

Data available on request to ale_pesce83@yahoo.it.
